# Optimization of kidney function in cardiac surgery patients with intra-abdominal hypertension: expert opinion

**DOI:** 10.1186/s13741-024-00416-5

**Published:** 2024-07-12

**Authors:** Vanessa Moll, Ashish K. Khanna, Andrea Kurz, Jiapeng Huang, Marije Smit, Madhav Swaminathan, Steven Minear, K. Gage Parr, Amit Prabhakar, Manxu Zhao, Manu L. N. G. Malbrain

**Affiliations:** 1 Department of Anesthesiology, Division of Critical Care Medicine, University of Minnesota, Minneapolis, MN USA; 2 Department of Anesthesiology, Division of Critical Care Medicine, Emory School of Medicine, Atlanta, GA USA; 3 Wake Forest University School of Medicine, Atrium Health Wake Forest Baptist Medical Center, Winston-Salem, NC USA; 4 Perioperative Outcomes and Informatics Collaborative (POIC), Winston-Salem, NC USA; 5 Outcomes Research Consortium, Cleveland, OH USA; 6 Departments of General Anesthesiology and Outcomes Research, Anesthesiology Institute, Cleveland Clinic, Cleveland, OH USA; 7 Department of Anesthesiology, Emergency Medicine and Intensive Care Medicine, Medical University Graz, Graz, Austria; 8 Department of Anesthesiology and Perioperative Medicine, University of Louisville, Louisville, KY USA; 9 Department of Critical Care, University Medical Center Groningen, University of Groningen, Groningen, Netherlands; 10 Department of Anesthesiology, Duke University Medical Center, Durham, NC USA; 11 Department of Anesthesiology, Cleveland Clinic Florida, Weston Hospital, Weston, FL USA; 12 Department of Anesthesiology and Critical Care Medicine, George Washington University School of Medicine and Health Sciences, Washington, DC USA; 13 Department of Anesthesiology, Cedars-Sinai Medical Center, Los Angeles, CA USA; 14 First Department of Anaesthesiology and Intensive Therapy, Medical University Lublin, Lublin, Poland; 15 Medical Data Management, Medaman, Geel, Belgium; 16 International Fluid Academy, Lovenjoel, Belgium

**Keywords:** Intra-abdominal pressure, Acute kidney injury, Monitoring, Prevention, Management, Cardiac surgery, Continuous intra-abdominal pressure, Abdominal-perfusion pressure

## Abstract

Cardiac surgery-associated acute kidney injury (CSA-AKI) affects up to 42% of cardiac surgery patients. CSA-AKI is multifactorial, with low abdominal perfusion pressure often overlooked. Abdominal perfusion pressure is calculated as mean arterial pressure minus intra-abdominal pressure (IAP). IAH decreases cardiac output and compresses the renal vasculature and renal parenchyma. Recent studies have highlighted the frequent occurrence of IAH in cardiac surgery patients and have linked the role of low perfusion pressure to the occurrence of AKI. This review and expert opinion illustrate current evidence on the pathophysiology, diagnosis, and therapy of IAH and ACS in the context of AKI.

**Supplementary Information:**

The online version contains supplementary material available at 10.1186/s13741-024-00416-5.

## Introduction

Cardiac surgery carries a substantial risk of acute kidney injury (CSA-AKI), with an incidence as high as 42% during the peri-operative period (Wu et al. 2019). Subsequently, up to 5% of patients require ongoing kidney replacement therapy (Vandenberghe et al. 2016; Machado et al. 2014). CSA-AKI is linked to higher perioperative and long-term mortality, extended ICU and hospital stay, and increased healthcare costs (Hobson et al. 2009; Ortega-Loubon et al. 2016; Srivastava et al. 2012). Furthermore, about 25% of patients with AKI develop chronic kidney disease (CKD) within 3 years (Horne et al. 2017).

AKI is diagnosed using the Kidney Disease Improving Outcomes (KDIGO) criteria, which incorporate serum creatinine (sCr) and urine output (UO) threshold values (Nadim 2018; Khwaja 2012). Various factors contribute to CSA-AKI, including patient-specific factors like comorbidities and age and pre-, intra-, and postoperative factors. Among these are neurohumoral activation, embolic phenomena, systemic inflammatory response, direct nephrotoxicity, and hemodynamic compromise (Milne et al. 2022).

Intra-abdominal hypertension (IAH), often overlooked in cardiac surgery patients, has been identified as an independent risk factor for postoperative AKI (Sun et al. 2021; Dalfino et al. 2013; Kopitkó et al. 2019). The inclusion of continuous intra-abdominal pressure (CIAP) in an AKI prediction algorithm allowed prognostication of AKI with higher precision and recall compared to the use of UO alone and up to 28 h before the event (Prabhakar et al. 2021). IAH, frequent in critically ill patients (Reintam Blaser et al. 2019; Murphy et al. 2018; Iyer et al. 2014), is associated with increased morbidity and mortality (Reintam Blaser et al. 2019; Murphy et al. 2018; Malbrain et al. 2014). In cardiac surgery, IAH has been described during the first 24–48 h postoperatively, with an incidence ranging from 46 to 83% (Dalfino et al. 2013; Iyer et al. 2014; Czajkowski and Dabrowski 2006; Dabrowski 2007; Xu et al. 2015; Dabrowski and Rzecki 2009; Kılıç et al. 2020; Tyson and Efthymiou 2021; Mazzeffi et al. 2016; Nazer et al. 2019; Smit et al. 2016; Ramser et al. 2021; Richer-Séguin et al. 2021). A recent analysis using CIAP monitoring revealed a persistent increase, with 1 in 5 patients experiencing ≥ 10 consecutive hours with an IAP > 20 mmHg in the first 48 h post-cardiac surgery (Khanna et al. 2022).

### Pathophysiology of AKI in association with IAH

The relationship between IAH and AKI involves a complex and multifactorial process. Elevated IAP causes direct compression of renal blood vessels reducing renal perfusion and oxygen delivery, leading to a decline in glomerular filtration (Doty et al. 2000; Mohmand and Goldfarb 2011). This triggers the renin–angiotensin–aldosterone hormone system (RAAS) signaling cascade, causing vasoconstriction and sodium and water retention. RAAS activation is an adaptive mechanism to maintain blood pressure, but angiotensin II and aldosterone may lead to a further decline in renal blood flow due to their vasoconstrictive effects on systemic vasculature (Devarajan 2005). IAH also induces an inflammatory response that may contribute to AKI (Maluso et al. 2016; Villa et al. 2016). In patients with advanced decompensated heart failure (HF), elevated IAP is frequent (Reintam Blaser et al. 2019), forming part of the cardio-abdominal-renal syndrome (CARS) concept (Verbrugge et al. 2013). Congestive HF can result in elevated IAP due to several potential mechanisms, such as ascites, bowel edema, ileus, and abdominal wall anasarca. Splanchnic venous congestion is associated with organ dysfunction (Beaubien-Souligny et al. 2020; Iida et al. 2016), likely through decreased renal perfusion pressure, further exacerbated by external capsular pressure from IAP. Increased IAP affects cardiovascular dynamics, compromising cardiac output (CO) and renal function. Elevated IAP leads to intra-abdominal and intrathoracic blood vessel compression, compromising macro- and microvascular blood flow (Cheatham 2009). Oliguria is one of the earliest signs of increased IAP (Mohmand and Goldfarb 2011; Malbrain 2005). Venous backpressure, measured either by central venous pressure (CVP) (Mullens et al. 2009) or Doppler indices (Beaubien-Souligny et al. 2020)and IAP (Mullens et al. 2008; Mullens et al. 2008), leads to venous congestion within the kidneys. Venous congestion impairs the normal venous outflow and further compromises renal blood flow. An increase in IAP above 15 mmHg further impairs microcirculation, and IAP > 25 mmHg critically reduces renal circulation. These circulatory changes correspond to a decrease in abdominal perfusion pressure (APP) (Sui et al. 2016). The impact of CVP and IAP reflects the importance of considering organ perfusion pressure instead of focusing only on inflow pressure. Mean perfusion pressure (MPP) is the difference between MAP and CVP or IAP (whichever is higher); and low MPP has been associated with organ system injury, including AKI (Panwar et al. 2020; Ostermann et al. 2017). However, CVP may be of limited value in several patient populations, and IAP may be a more specific marker for resistive abdominal forces. The Abdominal Compartment Society (WSACS, formerly known as the World Society of the Abdominal Compartment Syndrome) incorporated IAP into the equation for abdominal perfusion pressure (APP) to estimate the adequacy of renal perfusion (Kirkpatrick et al. 2013): APP = MAP-IAP. Low APP has been associated with poor patient outcomes, including higher rates of CSA-AKI (Wise et al. 2016; Dang et al. 2023). Even brief exposures to systemic hypotension, defined as a mean arterial pressure (MAP) less than 65 mmHg in critically ill patients, are associated with a significant increase in the risk of organ system injury, particularly AKI (Maheshwari et al. 2018). Individualized blood pressure targets are difficult to determine as they consist of a balance between driving force and resistive forces. A target MAP of 65 mmHg may not be adequate in critically ill patients due to a lack of perfusion pressure measurements. There is no simple protective target MAP in critically ill patients (Asfar et al. 2018).

Management of IAH and prevention of AKI involve strategies to reduce intra-abdominal pressure, optimize fluid balance, and maintain adequate renal perfusion. Early recognition and intervention are critical to improving outcomes in patients at risk of developing IAH and AKI.

Patients undergoing cardiac surgery are especially prone to developing high IAP; however, IAH remains underdiagnosed and -treated. We sought to create a novel stepwise algorithm incorporating continuous IAP and UO to prevent and manage AKI in cardiac surgery patients at risk for or with concomitant IAH.

## Definitions

### Abdominal hypertension

IAP, the steady-state pressure within the abdominal cavity (Kirkpatrick et al. 2013), is crucial for defining IAH and abdominal compartment syndrome (ACS). IAH is a sustained pathological elevation in IAP ≥ 12 mmHg, while ACS combines sustained IAP > 20 mmHg with new organ dysfunction/failure. IAH is graded, while ACS is an all-or-nothing phenomenon (Malbrain et al. 2006). In healthy adults, IAP is approximately 5–7 mmHg and 10 mmHg in critically ill adults.

Palpation-based clinical assessment of IAP is unreliable, necessitating standardized measurement techniques, such as intravesicular measurement, considered the gold standard (Ortega-Loubon et al. 2016). WSACS consensus definitions state that IAP should be expressed in mmHg and measured at end-expiration in the complete supine position after ensuring that abdominal muscle contractions are absent and with the transducer zeroed at the level of the midaxillary line at the level of the iliac crest. The reference standard for intermittent IAP measurement is via the bladder with a maximal instillation volume of 25 ml sterile saline (Malbrain et al. 2006).

### Acute kidney injury

The Kidney Disease Improving Global Outcomes (KDIGO) guidelines provide widely accepted consensus definitions for diagnosing and managing AKI (Kellum and Lameire 2013). sCr and UO are traditional biomarkers but have limitations. Even a small increase in sCr does not manifest until 50% of the renal glomerular filtration capacity is impaired. Hemodilution associated with cardiopulmonary bypass (CPB) and fluid resuscitation can mitigate any injury-related rise in sCr, especially in patients with concomitant cardiac or liver dysfunction (Jin et al. 2021), and in these patients, even small elevations in sCr can indicate advanced renal dysfunction (Nadim 2018). Further, sCr can be impacted by several non-renal factors, such as muscle mass, hydration, and medications that block tubular creatinine secretion (Kashani et al. 2020). Using the sCr criterion only underestimates AKI incidence (Koeze et al. 2017; Vaara et al. 2016). However, AKI by the UO alone is more prevalent but remains linked to higher mortality, particularly at higher AKI stages (Kellum and Lameire 2013; Vincent et al. 2020). While not a specific marker, UO can detect AKI earlier than sCr, with one study suggesting an AKI diagnosis 11 h earlier (Koeze et al. 2017; Dennen and Parikh 2007). Most research on AKI fails to include the UO criterion of the AKI definition (Jin et al. 2017), likely because UO measurements show wide variation and inconsistency. Hourly monitoring performed manually can be time-consuming for nurses, who may check only when the collection bags need emptying, for example, at 6 h. Continuous UO measurements are a promising and valuable tool for the early detection of AKI in critically ill patients. A continuous trend of 24-h UO recordings would provide important information like the area under the curve, the slope of urine production, and the time below or above a certain threshold. This information cannot be recognized with current intermittent techniques; hence daily fluctuations will be missed. Therefore, continuous UO monitoring allows better assessment of diagnostic tests (like the furosemide stress test) and responses to medical management.

While sCr and UO are markers of kidney function, they do not indicate early stress or injury of tubular cells within the nephron. Given these limitations, new biomarkers for the early diagnosis and risk stratification of CSA-AKI have been proposed (Ostermann et al. 2020) and included in the cardiac Enhanced Recovery After Surgery (ERAS) guidelines (Brown et al. 2003). The most studied and only FDA-approved urinary biomarkers in cardiac surgery patients are the combination of tissue inhibitor of metalloproteinases-2 [TIMP-2] and insulin growth factor-binding protein 7 [IGFBP7] using a cut-off value of [TIMP-2]•[IGFBP7] ≥ 0.3 (Zarbock et al. 2021; , Meersch et al. 2017). While this opinion paper focuses on the postoperative period, the authors recommend evaluating aortic atherosclerotic plaques with epiaortic ultrasound to guide CPB cannulations and minimize manipulation in advanced atherosclerosis cases. Moderate to severe aortic atherosclerotic burden, measured through epiaortic ultrasound, is associated with an increased risk of CSA-AKI (Iribarne et al. 2020).

## Medical management to optimize kidney function in cardiac surgery patients

Reducing CSA-AKI risk can significantly impact long-term patient outcomes and healthcare costs. Severe CSA-AKI is reported to the Society of Thoracic Surgeons (STS), and recently, AKI has been defined as a quality measure by the Centers for Medicare and Medicaid Services. There is a multidisciplinary drive aimed at improving patient outcomes through the standardization of care for CSA-AKI prevention: the Acute Disease Quality Initiative (ADQI) Consensus Statement (Nadim 2018), the ERAS Cardiac Society Guidelines (Engelman et al. 2019)and the Society of Thoracic Surgeons/Society of Cardiovascular Anesthesiologists/American Society of Extracorporeal Technology Clinical Practice Guidelines for the Prevention of Adult Cardiac Surgery-Associated Acute Kidney Injury (Brown et al. 2023). While all these guidelines incorporate the KDIGO recommendations, adherence to the kidney care bundle remains very low in cardiac surgery patients, with the full care bundle applied to only 0.4% of patients. As more studies define the role of APP in CSA-AKI (Dalfino et al. 2013; , Richer-Séguin et al. 2021; , Khanna et al. 2022), the addition of routine IAP monitoring postoperatively to guide perfusion pressures might be helpful.

We propose a protocol emphasizing KDIGO and the Abdominal Compartment Society (formerly known as the World Society of Abdominal Compartment Syndrome, WSACS) guidelines (Malbrain et al. 2006; , Kellum and Lameire 2013; , Kirkpatrick et al. 2017). This management strategy optimizes kidney perfusion and function in patients undergoing cardiac surgery. The algorithm follows a stepwise approach with a gradual increase in diagnostic tools and supportive management optimizing kidney function and IAP (Table1, Supplement, Supplemental Figure S1). Each step builds on the previous. If a patient, for example, is in AKI stage 2, steps 0 to 3 should be considered. If patients do not display IAH, the WSACS recommended steps to manage IAH, such as improving abdominal compliance and evacuating intraluminal and intra-abdominal contents, should be omitted.

**Table 1:** Stepwise algorithm incorporating continuous IAP and UO for preventing and managing AKI in cardiac surgery patients at risk for or with concomitant IAH

	Step 0	Step 1	Step 2	Step 3	Step 4	Step 5
KDIGO AKI staging using creatinine criteria	0	0	1	2	3	3
KDIGO AKI staging using UO criteria	–	AKI stage 1	AKI stage 1	AKI stage 2	AKI stage 3	AKI stage 3
Staging Criteria						
IAP (mmHg) (WSACS)	IAP < 10 mmHg – 2 consecutive measurements – 6 to 8 h apart	IAP 10–12 mmHg – 2 consecutive measurements – 4 to 6 h apart	IAP 12–15 mmHg – 2 consecutive measurements – 4 to 6 h apart	IAP 16–20 mmHg – 2 consecutive measurements – 4 to 6 h apart – Initiate CIAP	IAP 21–25 mmHg – 2 consecutive measurements – 2 to 4 h apart – CIAP	IAP > 25 mmHg – 2 consecutive measurements – 1 to 2 h apart – CIAP
CIAP (mmHg)	CIAP < 10 mmHg for 2 consecutive hours	CIAP 10–12 mmHg for 2 consecutive hours	CIAP 12–15 mmHg for 2 consecutive hours	CIAP 16–20 mmHg for 2 consecutive hours	CIAP 21–25 mmHg for 2 consecutive hours	CIAP > 25 mmHg for 2 consecutive hours
APP (mmHg)	APP > 65 mmHg – 2 consecutive measurements – 4 to 6 h apart	APP < 65 mmHg – 2 consecutive measurements – 4 to 6 h apart	APP < 60 mmHg – 2 consecutive measurements – 4 to 6 h apart	APP < 55 mmHg – 2 consecutive measurements – 4 to 6 h apart	APP < 50 mmHg – 2 consecutive measurements – 4 to 6 h apart	APP < 45 mmHg – 2 consecutive measurements – 4 to 6 h apart
CAPP (mmHg)	CAPP > 65 mmHg for 2 consecutive hours	CAPP < 65 mmHg for 2 consecutive hours	CAPP < 60 mmHg for 2 consecutive hours	CAPP < 55 mmHg for 2 consecutive hours	CAPP < 50 mmHg for 2 consecutive hours	CAPP < 45 mmHg for 2 consecutive hours
UO (KDIGO)	UO > 0.5 ml/kg/hr for 6–12 h	UO < 0.5 ml/kg/h for 6–12 h	UO < 0.5 ml/kg/h for 6–12 h	UO < 0.5 ml/kg/h > 12 h	UO < 0.3 ml/kg/h > 24 h OR anuria > 12 h	UO = 0 ml/kg/h (anuria) > 24 h
CUO	CUO > 0.5 ml/kg/h	CUO < 0.5 ml/kg/h for 2–4 h	CUO < 0.5 ml/kg/h for 2–4 h	CUO < 0.5 ml/kg/h > 4 h	CUO < 0.3 ml/kg/h > 8 h OR anuria > 4 h	CUO = 0 ml/kg/h (anuria) > 8 h
Creatinine (KDIGO)	sCr < 1.5 times baseline	sCr < 1.5 times baseline	sCr 1.5–1.9 × baseline (within 7 days) Or ≥ 0.3 mg/dl increase (within 48 h)	sCr 2.0–2.9 × baseline	sCr 3.0 × baseline OR Increase in sCr to ≥ 4.0 mg/dl (with ≥ 0.3 mg/dl increase within 48 h or 1.5 × baseline) OR Initiation of kidney replacement therapy	sCr 3.0 × baseline OR Increase in sCr to ≥ 4.0 mg/dl (with ≥ 0.3 mg/dl increase within 48 h or 1.5 × baseline) OR Initiation of kidney replacement therapy
Kidney biomarkers ([TIMP2]•[IGFBP7] predict risk for AKI and do not correlate with sCr measurements at the time taken) (Kashani et al. 2013; , Meersch et al. 2014)	Low risk result < 0.3	Increased risk 0.3 ≤ Result ≤ 2	High risk result > 2.0			
Protocol steps						
		Same as the previous step Plus				
Monitoring	- Bladder catheter, arterial catheter (remove when stable parameters) - Close monitoring of sCr (1 × /day), - UO (1 × /2 h) or hourly while in ICU - IAP and APP (2 × /day) - Fluid balance and body weight (1 × /day) - Obtain baseline BIA - Optimization of hemodynamics – MAP > 65 mmHg – APP > 60 mmHg	- Beat-to-beat alternatives or other noninvasive hemodynamic monitoring - UO (1 × /2 h) or hourly while in ICU - IAP and APP (4 × /day) or CIAP and CAPP - Fluid balance (2 × /day) - Daily BIA	- UO (1 × /h) or continuously - IAP and APP (6 × /day) or CIAP and CAPP - Central venous catheter - Uncalibrated CO monitoring with optimization of hemodynamics – SVV < 12–15% – PPV < 12–15%	- Calibrated CO-monitoring with optimization of hemodynamics – CI > 2.5 – CVP_TM_ = CVP_ee_–IAP/2	Same as the previous step	If IAP is persistently increased above 20–25 mmHg with new-onset organ dysfunction or failure or worsening kidney function, and refractory under medical management: surgical abdominal decompression with temporary abdominal closure (e.g., VAC dressing) should be considered
Drug actions	- Avoidance of hydroxyethyl starch, gelatin, and chloride-rich solutions - Start ERAS protocol when available - Judicious use of balanced crystalloids - Maintain blood glucose 80–180 mg/dl	- Consideration of alternatives to radiocontrast agents within first 72 h after surgery or shock onset - Discontinuation and avoidance of any nephrotoxic substances for 72 h - Transfusion if hemoglobin < 7 g/dl or hematocrit < 21%	- Urine analysis - IVF and vasopressors for (C)APP > 65 mmHg - Goal SpO2 more than 92–96% with PaO2 70–100 mmHG	- Multidisciplinary Acute Kidney Response Team and/or nephrology consult - Inotropes as needed consider balanced use of catecholamines and vasopressin with early introduction of angiotensin II when indicated (Coulson et al. 2022)	Same as the previous step	
Optimize fluid administration (POCUS)	- Avoid excessive fluid resuscitation - Avoid normal saline (NaCl 0.9%) - Apply goal-directed fluid resuscitation when indicated - Only give fluids when needed (state of shock DO2/VO2 imbalance, lactate > 3 mmol, BE < − 8)	POCUS: Transthoracic echocardiography, IVC to assess for volume status/fluid responsiveness if indicated - Aim for zero to negative daily fluid balance - Initiate late conservative fluid management when indicated	POCUS: Transthoracic echocardiography, IVC to assess for volume status/fluid responsiveness if indicated - Fluids are guided by uncalibrated CO monitoring in combination with dynamic CVP - Assessment for fluid responsiveness: end-expiratory occlusion or fluid challenge can be used - Prefer colloids over crystalloids - Consider the furosemide stress test	POCUS: Transthoracic echocardiography, IVC to assess for volume status/fluid responsiveness if indicated Daily/PRN Venous Excess Ultrasonography Score (VExUS), renal resistive index (RRI) - Fluid removal through judicious diuresis once the patient is stable - Combination therapy of diuretics can be used	- Consider hemodialysis or CVVH with net ultrafiltration	
Improve abdominal wall compliance (WSACS guidelines)	- Optimize pain control - Avoid morphine analogs - Consider regional anesthesia techniques - Avoid tight abdominal dressings	POCUS: Abdominal ultrasound - Control fever - Remove constrictive abdominal dressings if present	POCUS: Abdominal ultrasound - Consider reverse Trendelenburg position - Avoid HOB 45° especially if BMI > 25–30	POCUS: Abdominal ultrasound Consider sedation, intubation, and mechanical ventilation if not yet ventilated - Lung protective ventilation – Best PEEP (cmH20) = IAP (mmHg) – Pplat_TM_ = Pplat + 7–(IAP × 0.7) - Trial of bolus of neuromuscular blockers	- Deep sedation - Consider continuous infusion neuromuscular blocker	
Evacuate intra-luminal and intra-abdominal contents (WSACS guidelines)	- Insert nasogastric tube when indicated - Ensure drains (when inserted) evacuate properly	POCUS: Abdominal ultrasound - Initiate gastroprokinetics (i.e., cisapride) - Initiate colonoprokinetics (i.e., erythromycin)	POCUS: Abdominal ultrasound - Minimize enteral nutrition speed - Initiate stool protocol if not active yet - Perform abdominal computed tomography without contrast to identify lesions, ascites, or collections if suspected	POCUS: Abdominal ultrasound - Administer enemas or consider colonoscopic decompression (Ogilvie) - Consider percutaneous POCUS-guided drainage of collections if present	POCUS: Abdominal ultrasound - Stop enteral nutrition - Consider CT-guided or surgical evacuation of lesions	

*ACEI* angiotensin-converting-enzyme inhibitors, *ARB* angiotensin II receptor blockers, *AKRT* acute kidney response team, *APP* abdominal perfusion pressure, *BIA* bio-electrical impedance analysis, *BMI* body mass index, *BW* body weight, *CAPP* continuous abdominal perfusion pressure, *CI* cardiac index, *CIAP* continuous intra-abdominal pressure, *CO* cardiac output, *CSA* cross-sectional area, *CT* computed tomography, *CUO* continuous urine output, *CVP* central venous pressure, *CVVH* continuous venovenous hemofiltration, *ee* end-expiratory, *EEO* end-expiratory occlusion, *ERAS* enhanced recovery after surgery, *FST* furosemide stress test, *GFR* glomerular filtration rate, *GUTS* gastrointestinal and urinary tract sonography, *HOB* head of bed, *IAP* intra-abdominal pressure, *ICU* intensive care unit, *IVC* inferior vena cava, *KDIGO* Kidney Disease: Improving Global Outcomes, *LCFM* late conservative fluid management, *LVOT* left ventricular outflow tract, *NMB* neuromuscular blockers, *NSAID* non-steroidal anti-inflammatory drugs, *PEEP* positive end-expiratory pressure, *PLR* passive leg raising, *POCUS* point of care ultrasound, *Pplat* alveolar plateau pressure, *PPV* pulse pressure variation, *SVV* stroke volume variation, *TDI* tissue doppler imaging, *TEE* transesophageal echocardiography, *TM* transmural, *TTE* transthoracic echocardiography, *UF* ultrafiltration, *UO* urine output, *US* ultrasound, WSACS: the Abdominal Compartment Society

## Step 0: baseline evaluation

### Parameters

Baseline intermittent IAP values are normal (< 10 mmHg) for two consecutive measurements taken 4 to 6 h apart or CIAP < 10 mmHg for 2 h, sCr is not increased (< 1.5 × baseline creatinine), and UO is normal (> 1 ml/kg/h for 6 to 12 h or continuous UO (CUO) > 1 ml/kg/h for 2 h). The intermittent APP values are > 65 mmHg for 2 consecutive measurements taken 4 to 6 h apart or continuous APP (CAPP) > 65 mmHg for 2 h. Urinary biomarkers of renal stress, such as [TIMP-2] × [IGBF7], can be performed every 6 h after cardiac surgery to assess the risk for AKI. A baseline body weight should be measured pre-operatively and daily.

### Monitoring of organ function

In order to optimize kidney and organ function, the patient is equipped with a Foley catheter, an arterial catheter, or other noninvasive beat-to-beat alternatives to monitor MAP. Close monitoring of sCr (1 × /day), UO (1 × /4 h or hourly while in ICU), IAP (2 × /day), and daily and cumulative fluid balance (1 × /day) is performed. Obtaining a baseline BIA measurement is warranted to assess body fluid composition. Hemodynamics should be optimized by targeting MAP > 65 mmHg and (C)APP > 60 mmHg.

### Drug actions and optimization of fluid administration

Goal-directed hemodynamic resuscitation and ERAS protocols (Engelman et al. 2019; , Osawa et al. 2016; , Johnston et al. 2020) should be initiated. Fluid management should be guided by monitoring devices that can detect hypovolemia, assess the response to fluid infusion, and determine the need for fluid removal. Balanced crystalloids are recommended before considering colloids. Balanced crystalloid solutions are associated with a lower risk of AKI in critically ill patients than 0.9% saline (Hammond et al. 2020). This benefit is likely due to avoiding chloride-mediated vasoconstriction and chloride-associated metabolic acidosis (Bailey et al. 2015). Colloids are used widely as fluid bolus therapy as they are thought to provide a more sustained physiological response than a similar volume of crystalloid. Albumin has widely been used in cardiac surgery. The purported protective effects of albumin on the endothelial glycocalyx, microvascular integrity, reduced platelet consumption, and prevention of AKI are mainly described in preclinical studies, small clinical trials, or non-cardiac surgery critically ill patient populations. In the ALBICS trial, albumin was effective for volume expansion but did not have superior effects on morbidity (Pesonen et al. 2022). The benefit of fluid balance was confirmed in the HAS FLAIR trial using 20% albumin (Wigmore et al. 2019). Recent guidelines recommend using albumin following crystalloid resuscitation in patients to avoid an excessive positive fluid balance (Xiang et al. 2023). Older hydroxyethyl starch (HES) solutions with large molecular sizes and high molar substitution negatively impacted kidney function (Zarychanski et al. 2013). In contrast, modern tetrastarch 6% HES 130/0.4–0.42 appears safe (Kopitkó 2023; Hydroxyethyl 2022). A large trial (Phoenics trial, NCT03278548, registered September 11, 2017) is currently investigating the impact of HES on kidney function during the perioperative period. The results might inform whether HES can be recommended. Data on gelatin use and AKI are limited, but observational studies suggest they may contribute to AKI through osmotic nephrosis (Moeller et al. 2016). Therefore, gelatins are also not recommended in AKI (Khwaja 2012; , Ostermann et al. 2019).

For edematous patients who are intravascularly dry, limited evidence suggests that resuscitation with hypertonic saline reduces perioperative complications, ICU days, and mortality (Pfortmueller and Schefold 2017). However, the daily maximum sodium and chloride load is a concern (Chatterjee and Garg 2024).

Blood glucose should be maintained between 80 and 180 mg/dl (Lazar et al. 2009).

### Improving abdominal wall compliance

While morphine analogs, especially fentanyl, may cause abdominal muscle contractions, thereby decreasing abdominal compliance (Malbrain et al. 2014), we cannot endorse the avoidance of these potent analgesics in the immediate postoperative phase. However, regional anesthesia techniques and epidural analgesia should be considered instead or as an adjunct when possible. Tight abdominal closure or constrictive dressings must be avoided when applicable.

### Evacuation of intra-luminal or intra-abdominal contents

According to the WSACS guidelines (Kirkpatrick et al. 2013), a nasogastric tube should be inserted when indicated. Ensure that chest or other drains (when inserted) evacuate properly.

## Step 1: prevention

### Parameters

IAP values measured intermittently are high normal (10–12 mmHg) for 2 consecutive measurements taken 4 to 6 h apart or CIAP 10–12 mmHg for 2 h, sCr remains less than 1.5 × the baseline, but UO is decreased (< 0.5 ml/kg/h for 6 to 12 h or CUO < 0.5 ml/kg/h for 2 to 4 h). Intermittent APP values are < 65 mmHg for 2 consecutive measurements taken 4 to 6 h apart or CAPP < 65 mmHg for 2 h. Kidney biomarkers are abnormal.

### Monitoring of organ function

Same as the previous step. Additionally, we recommend close monitoring of sCr (2 × /day), UO (1 × /2 h or hourly while in ICU or CUO), IAP and APP (4 × /day) (or CIAP and CAPP), fluid balance (2 × /day), BIA and body weight daily.

### Drug actions

Consider alternatives to radiocontrast agents in the first 72 h after surgery or shock onset. Avoid nephrotoxic substances for 72 h if clinically indicated (e.g., angiotensin-converting-enzyme inhibitors (ACEI) and angiotensin II receptor blockers (ARB), non-steroidal anti-inflammatory drugs (NSAIDs), vancomycin, aminoglycosides, and proton pump inhibitors). Balanced or buffered IV fluids and vasopressors (e.g., norepinephrine) should be titrated for MAP > 65 mmHg or (C)APP > 60 mmHg. Packed red blood cells should be transfused if serum hemoglobin < 7 g/dl or hematocrit < 21% (consider a higher threshold such as hemoglobin, 8 g/dl in case of active cardiac or respiratory comorbidities).

### Optimization of fluid administration

POCUS should be performed with baseline transthoracic echocardiography (TTE, critical care echocardiography) or transesophageal echocardiography (TEE) to check left ventricular and right ventricular systolic and diastolic functions. It is advocated to aim for zero to negative daily fluid balance and to initiate late conservative fluid management (Malbrain et al. 2018), defined as two consecutive negative daily fluid balances within the first week when indicated.

### Improving abdominal wall compliance

Abdominal wall thickness should be measured with POCUS in the setting of adequate analgesia and normothermia. It is advocated to remove constrictive abdominal (velcro) dressings when present.

### Evacuation of intra-luminal or intra-abdominal contents

If not performed previously, a nasogastric (NGT) and rectal tube can be inserted when indicated. POCUS can be used to confirm the NGT position and calculate the antral cross-sectional area. When indicated, initiate gastroprokinetics (cisapride or metoclopramide) and/or colonoprokinetics (erythromycin). Abdominal ultrasound can be performed to identify lesions or collections if suspected in combination with the gastrointestinal and urinary tract sonography (GUTS) protocol (Perez-Calatayud et al. 2018).

## Step 2: monitoring optimization

### Parameters

At this stage, the following parameters are met. The baseline intermittent IAP values are slightly increased (12–15 mmHg) for 2 consecutive measurements taken 4 to 6 h apart or CIAP 12–15 mmHg for 2 h. sCr is increased (sCr 1.5–1.9 × baseline (within 7 days) or ≥ 0.3 mg/dl increase (within 48 h)). UO is decreased (< 0.5 ml/kg/h for 6 to 12 h or CUO < 0.5 ml/kg/h for 2 to 4 h). The intermittent APP values are < 60 mmHg for 2 consecutive measurements taken 4 to 6 h apart or CAPP < 60 mmHg for 2 h. Kidney biomarkers are abnormal.

### Monitoring of organ function

Increase the monitoring frequency of UO (1 × /h), IAP, and APP (4 × /day) or CIAP and CAPP. If no previously indwelled pulmonary artery catheter, we suggest initiating CO monitoring targeting SVV < 12–15% or PPV < 12–15%.

### Drug actions

A urine analysis should be obtained (electrolytes, osmolarity, glomerular filtration rate (GFR)), and renal biomarkers checked. Target SpO_2_ is 92–96% and PaO_2_ of 70–100 mmHg.

### Optimization of fluid administration

POCUS of IVC and TTE should be performed with attention to the left ventricular outflow tract (LVOT). Calculate the tissue Doppler imaging (TDI) derived ratio of early diastolic mitral inflow velocity to early diastolic mitral annulus velocity (E/E’) to assess volume status, diastolic function, and response to fluids or diuresis (Vermeiren et al. 2015). Fluids are guided by calibrated or uncalibrated CO monitoring (Osawa et al. 2016; , Johnston et al. 2020; , Bernards et al. 2015; , Denault et al. 2020)either in combination with a positive EEO test (end-expiratory occlusion, with 5% increase CO), a (mini) fluid challenge (15% increase CI) (Malbrain et al. 2018), passive leg raise test (Monnet et al. 2016)or other means of critical care echocardiography. In ventilated patients, a tidal volume challenge might also be helpful (Wang et al. 2023). The furosemide stress test (FST) can be performed to assess kidney reserve (Chawla et al. 2013; , McMahon and Chawla 2021). Colloids (albumin 20% or 25%) or hypertonic solutions (NaCl 3%) are preferred in selected cases to minimize volume overload.

### Improving abdominal wall compliance

POCUS can be performed to detect whether there is any abdominal wall movement. The reverse Trendelenburg position should be considered if there are no contraindications (e.g., brain trauma or risk for aspiration). The head of bed elevation (HOB) > 30° position needs to be avoided, especially if BMI > 25–30, as HOB of 45° position can increase IAP by 5 to 15 mmHg (De Keulenaer et al. 2009).

### Evacuation of intra-luminal and intra-abdominal contents

Abdominal POCUS can be performed to assess the degree of bowel activity, dilation, or feces (Abu-Zidan and Cevik 2018). The enteral nutrition speed should be lowered, and a stool protocol activated if not already done. Abdominal computed tomography can be performed to identify lesions or collections if suspected.

## Step 3: organ support

### Parameters

There are moderately increased IAP values (15–20 mmHg) for 2 consecutive measurements taken 4 to 6 h apart or CIAP 15–20 mmHg for 2 h. sCr is increased (2–2.9 × baseline). UO is decreased (< 0.5 ml/kg/h for 12 h or CUO < 0.5 ml/kg/h for 4 h). The intermittent APP values are < 55 mmHg for 2 consecutive measurements taken 4 to 6 h apart or CAPP < 55 mmHg for 2 h.

### Monitoring of organ function

Monitoring continues as in the previous step. Additionally, we recommend inserting a central venous catheter and using calibrated CO-monitoring. Barometric preload indicators like CVP or pulmonary artery occlusion pressure (PAOP, wedge pressure) cannot be trusted. Both will erroneously be increased because of the abdomino-thoracic transmission of IAP (Malbrain et al. 2015). Consider measuring CIAP if available. Continue hemodynamic optimization and aim for CI > 2.5. Transmural CVP = CVPee–IAP/2 (ee, end-expiratory).

### Drug actions

Inotropes are titrated as needed in combination with balanced or buffered IV fluids and vasopressors. Catecholamines and vasopressin should be combined, and Angiotensin II introduced when indicated (Coulson et al. 2022). If available and not already informed, the multidisciplinary Acute Kidney Response Team or nephrology consult services should be contacted (Engelman et al. 2020; , Rizo-Topete et al. 2017). Additionally, we recommend consulting general surgery in case the patient deteriorates to ACS and needs surgical intervention/decompression.

### Optimization of fluid administration

POCUS can be used to assess renal, hepatic arterial, and portovenous blood flow, guiding fluid removal through judicious diuresis once the patient is hemodynamically stable. The venous congestion score (VExUS) is calculated in combination with the renal resistive index and should be continued daily. Indices of venous stasis such as common femoral vein Doppler or intrarenal venous flow examination may be observed in hypervolemia but also in impaired right ventricular function or conditions with elevated intrathoracic pressures (Denault et al. 2020; , Bhardwaj et al. 2023; , Beaubien-Souligny et al. 2018). Fluid removal can be achieved via combination therapy of diuretics (loop diuretics + acetazolamide + indapamide/metolazone) or the combination with albumin 20% (in hypo-proteinemic patients) or PAL treatment (PEEP plus ALBUMIN plus LASIX (Furosemide)) (Cordemans et al. 2012). Spironolactone should be avoided in patients with increased potassium. Recently the ADVOR trial showed a beneficial effect of adding acetazolamide, especially in patients with decompensated HF and increased base excess (Mullens et al. 2022).

### Improving abdominal wall compliance

Sedation, intubation, and mechanical ventilation (if not yet mechanically ventilated) should be considered. Lung protective ventilation strategy (Evans et al. 2021; , Regli et al. 2019) is recommended aiming for the best PEEP (cmH_2_0) equal to IAP (mmHg) and targeting transmural plateau pressure (Pplat) towards Pplat + 7 – (IAP × 0.7) and avoiding driving pressures above 14 cm H2O (Regli et al. 2019). A trial with bolus neuromuscular blockers can be considered (De Laet et al. 2007). It is recommended to consult an abdominal surgeon early if the patient's condition worsens.

### Evacuation of intra-luminal and intra-abdominal contents

If not otherwise documented, POCUS can identify the need for enema therapy. Rectal enemas can be administered, or colonoscopic decompression considered (in the case of Ogilvie syndrome). The use of percutaneous POCUS-guided drainage of collections should be considered. The speed of enteral nutrition should be tapered, and brittle or trophic feeding can be considered.

## Step 4: de-escalation

### Parameters

Intermittent IAP values are severely increased (20–25 mmHg) for 2 consecutive measurements taken 4 to 6 h apart or CIAP 20–25 mmHg for 2 h. sCr is increased (3.0 × baseline or increase in sCr to ≥ 4.0 mg/dl, kidney replacement therapy was initiated). UO is decreased (< 0.3 ml/kg/h for > 24 h or anuria > 12 h or CUO < 0.3 ml/kg/h for > 8 h or anuria for > 4 h). The intermittent APP values are < 50 mmHg for 2 consecutive measurements taken 4 to 6 h apart or CAPP < 50 mmHg for 2 h.

### Monitoring

Same as the previous step (step 3).

### Drug actions

IV fluids and vasopressors are de-escalated or discontinued whenever possible. Diuretics are started when appropriate (e.g., positive cumulative fluid balance, increased BIA volume excess, venous congestion).

### Optimization fluid administration

Daily calculations of the venous congestion score (VExUS) and the renal resistive index are continued. De-resuscitation is considered with the use of hemodialysis or continuous venovenous hemofiltration (CVVH) with net ultrafiltration is considered.

### Improving abdominal wall compliance

Deep sedation can be considered and combined with neuromuscular blockers in continuous infusion.

### Evacuation of intra-luminal and intra-abdominal contents

Enteral nutrition should be stopped temporarily. US or computer tomography can be used to follow up or determine the evolution of fluid and/or blood collections. Surgical evacuation of lesions should be considered when appropriate.

### Immediate action

ACS is diagnosed if IAP is persistently above 20 mmHg with new-onset organ dysfunction or failure like worsening kidney function. Consultation with an abdominal surgeon is recommended if not already alerted. Medical management with optimization of organ function and fluid management, improvement of abdominal wall compliance, and evacuation of intra-abdominal contents need to be escalated. If the patient deteriorates or fails to improve with maximum medical management, surgical abdominal decompression with temporary abdominal closure (e.g., VAC dressing) should urgently be considered.

## Step 5: decompression

### Parameters

Intermittent IAP values are increased > 25 mmHg for 2 consecutive measurements taken 4 to 6 h apart or CIAP > 25 mmHg for 2 h, sCr is increased (3.0 × baseline or increase in sCr to ≥ 4.0 mg/dl, kidney replacement therapy was initiated). UO has stopped (0 ml/kg/h for > 24 h or CUO = 0 ml/kg/h for > 8 h). The intermittent APP values are < 45 mmHg for 2 consecutive measurements taken 4 to 6 h apart or CAPP < 45 mmHg for 2 h.

### Immediate action

ACS is diagnosed if IAP is persistently above 20 mmHg with new-onset organ dysfunction or failure like worsening kidney function. If the patient fails to improve with maximum medical management, surgical abdominal decompression with temporary abdominal closure (e.g., VAC dressing) should urgently be considered.

## Discussion

IAH is frequently diagnosed in critically ill patients, where its occurrence is well-documented and associated with significant morbidity and mortality. However, in cardiac surgery patients, IAH remains often underdiagnosed and poorly understood. Despite the prevalence of conditions that predispose cardiac surgery patients to IAH—such as fluid overload, massive transfusion, and CPB, there is a lack of routine monitoring and awareness among healthcare providers in this specific population. This gap in recognition can lead to a delay in diagnosis and treatment and potentially worse patient outcomes. This proposed algorithm serves as a practical guide for clinicians, highlighting the importance of continuous monitoring, personalized interventions, and multidisciplinary collaboration in optimizing kidney function in patients with IAH. Additionally, the low adherence to kidney care bundles in cardiac surgery patients (Massoth et al. 2003) underscores the need for improved implementation. The stepwise approach incorporates the KDIGO and WSACS guidelines with the goal of preventing and managing CSA-AKI as it relates to increased IAP.

The impact of elevated IAP extends beyond the abdominal cavity, affecting multiple organ systems. Notably, elevated IAP and low APP have an early and significant effect on the renal system, making them crucial modifiable factors for preventing and alleviating AKI. Venous back pressure, as measured by CVP (Mullens et al. 2009), Doppler indices (Iida et al. 2016), and IAP (Mullens et al. 2008; , Mullens et al. 2008; , Bachmann et al. 2022), worsens kidney function. Therefore, a comprehensive consideration of organ perfusion pressure, accounting for back pressure on the venous side rather than solely focusing on inflow pressure, is essential. MPP and APP play pivotal roles in assessing and predicting the progression of organ system injury (Reintam Blaser et al. 2019; Murphy et al. 2018; Iyer et al. 2014).

Patients with advanced decompensated HF often exhibit elevated IAP (Mullens et al. 2008; Rubio-Gracia et al. 2020), indicating a major pathophysiological role during HF decompensations (Afsar et al. 2016; , Husain-Syed et al. 2021) and contributing CARS. IAH can reduce CO (Bloomfield et al. 1996; , Ridings et al. 1995), leading to a range of cardiovascular changes. IAH leads to a cephalad movement of the diaphragm, directly compressing the heart with a reduction of the right and left ventricle end-diastolic volumes. Cardiac preload decreases due to decreased venous return from the abdomen, and systemic afterload increases due to the direct compression of vascular beds and the activation of the RAAS pathway (Bloomfield et al. 1997; , Gudmundsson et al. 2002). Experimental induction of IAH has been shown to decrease CO and stroke volume while increasing vascular resistance (Ivankovich et al. 1975). Elevated IAP increases blood pressure in the pulmonary circulation and PAOP (Ridings et al. 1995). Supplemental Figure S1 illustrates the well-established cardiac dysfunction resulting from IAH (Dabrowski et al. 2023).

The rapid changes in cardiac perfusion and cardiovascular depression following IAH pose a significant risk factor for ACS and poor outcomes. The decrease in CO related to IAH reduces microcirculatory perfusion, particularly in vulnerable organs such as the kidney, small bowel, and colon mucosa (Olofsson et al. 2009). Acute organ hypoperfusion, combined with an inflammatory response, elevates the risk of organ insufficiency, creating a potentially vicious cycle leading to ACS. Inappropriate fluid administration, including fluid overload and positive perioperative fluid balance, is identified as a significant risk factor for IAH and ACS (Duchesne et al. 2015). Postoperative ACS occurs in about 1% of patients after cardiopulmonary bypass and carries a high mortality rate of 57% (Ramser et al. 2021). Eliminating IAH in the postoperative period is crucial to preventing cardiac dysfunction (Panwar et al. 2020), optimizing cardiac and kidney function, and potentially reducing CSA-AKI.

Considering the myriad issues that can arise from IAH after cardiac surgery, particularly kidney dysfunction, the presented management strategy aims to prevent and treat significant kidney injury in this patient population. CUO and IAP measurements play instrumental roles, and the use of novel biomarkers further enhances the implementation of this strategy. The authors highlight the urgent need for robust scientific evidence to support the recommendations presented in this review. To ensure these guidelines are widely adopted and validated, it is crucial to undertake randomized controlled trials, observational studies, and retrospective analyses.

## Conclusion

CSA-AKI stands as a significant contributor to postoperative morbidity and mortality. In this context, IAH emerges as a potential factor in the development of CSA-AKI, independently linked to heightened morbidity and mortality. Beyond adhering to the recommended KDIGO kidney care bundle, clinicians should incorporate monitoring IAP and addressing IAH as preventive measures against the onset of kidney and organ dysfunction. Optimizing patient care throughout the postoperative period remains pivotal in mitigating the burden of CSAA-AKI.

### Supplementary Information

Supplementary Material 1: Supplementary Figure S1. Assessment of fluid responsiveness with functional hemodynamics. Supplementary Figure S2. Assessment of abdominal wall compliance (Cab). Supplementary Table S1. When to monitor abdominal wall compliance? Supplementary Table S2. Indicators for decreased abdominal wall compliance. Supplementary Table S3. Indicators for increased abdominal wall compliance.

## Data Availability

N/A.

## Abbreviations

ACEI: Angiotensin-converting-enzyme inhibitors
ACS: Abdominal compartment syndrome
ADQI: Acute Disease Quality Initiative
AKI: Acute kidney injury
APP: Abdominal perfusion pressure
ARB: Angiotensin II Receptor Blockers
BIA: Bio-electrical impedance analysis
BMI: Body mass index
CAPP: Continuous abdominal perfusion pressure
CARS: Cardio-abdominal-renal syndrome
CIAP: Continuous intra-abdominal pressure
CKD: Chronic kidney disease
CO: Cardiac output
CPB: Cardiopulmonary bypass
CSA-AKI: Cardiac surgery-associated acute kidney injury
CUO: Continuous urine output
CVP: Central venous pressure
CVVH: Continuous venovenous hemofiltration
E/E’: Early diastolic mitral inflow velocity to early diastolic mitral annulus velocity
Ee: End-expiratory
EEO: End-expiratory occlusion
ERAS: Enhanced Recovery After Surgery (ERAS)
FST: Furosemide stress test
GUTS: Gastrointestinal and urinary tract sonography
HES: Hydroxyethyl starch
HF: Heart failure
HSS: Hypertonic sodium solutions
HOB: Head of bed elevation
IAH: Intra-abdominal hypertension
IAP: Intra-abdominal pressure
IGFBP-7: Insulin-like growth factor-binding protein-7
IVC: Inferior vena cava
KDIGO: Kidney disease: improving global outcomes
LVOT: Left ventricular outflow tract
LVOT VTI: Left ventricular outflow tract velocity time integral
MAP: Mean arterial pressure
MPP: Mean perfusion pressure
NGT: Nasogastric tube
PAL: PAL is defined as PEEP set at the level of IAP, Albumin 20%, followed by Lasix (Furosemide)
PLR: Passive leg raising test
POCUS: Point-of-care ultrasound
RRI: Renal resistive index
sCr: Serum creatinine
STS: Society of Thoracic Surgeons
SV: Stroke volume
TDI: Tissue Doppler imaging
TIMP-2: Tissue inhibitor of metalloprotease-2
UO: Urine output
VExUS: Venous Excess Ultrasound Score
WSACS: Abdominal Compartment Society (formerly known as the World Society of Abdominal Compartment Syndrome)
