# Correction: Optimization of kidney function in cardiac surgery patients with intra-abdominal hypertension: expert opinion

**DOI:** 10.1186/s13741-024-00444-1

**Published:** 2024-07-29

**Authors:** Vanessa Moll, Ashish K. Khanna, Andrea Kurz, Jiapeng Huang, Marije Smit, Madhav Swaminathan, Steven Minear, K. Gage Parr, Amit Prabhakar, Manxu Zhao, Manu L. N. G. Malbrain

**Affiliations:** 1 Department of Anesthesiology, Division of Critical Care Medicine, University of Minnesota, Minneapolis, MN USA; 2 Department of Anesthesiology, Division of Critical Care Medicine, Emory School of Medicine, Atlanta, GA USA; 3 Wake Forest University School of Medicine, Atrium Health Wake Forest Baptist Medical Center, Winston-Salem, NC USA; 4 Perioperative Outcomes and Informatics Collaborative (POIC), Winston-Salem, NC USA; 5 Outcomes Research Consortium, Cleveland, OH USA; 6 Departments of General Anesthesiology and Outcomes Research, Anesthesiology Institute, Cleveland Clinic, Cleveland, OH USA; 7 Department of Anesthesiology, Emergency Medicine and Intensive Care Medicine, Medical University Graz, Graz, Austria; 8 Department of Anesthesiology and Perioperative Medicine, University of Louisville, Louisville, KY USA; 9 Department of Critical Care, University Medical Center Groningen, University of Groningen, Groningen, Netherlands; 10 Department of Anesthesiology, Duke University Medical Center, Durham, NC USA; 11 Department of Anesthesiology, Cleveland Clinic Florida, Weston Hospital, Weston, FL USA; 12 Department of Anesthesiology and Critical Care Medicine, George Washington University School of Medicine and Health Sciences, Washington, DC USA; 13 Department of Anesthesiology, Cedars-Sinai Medical Center, Los Angeles, CA USA; 14 First Department of Anaesthesiology and Intensive Therapy, Medical University Lublin, Lublin, Poland; 15 Medical Data Management, Medaman, Geel, Belgium; 16 International Fluid Academy, Lovenjoel, Belgium

**Correction** **:** **Perioper Med 13, 72 (2024)**

**https://doi.org/10.1186/s13741-024-00416-5**

Following publication of the original article (Moll et al. 2024), the author reported an error in the affiliation order of the authors. This has been corrected and the original article (Moll et al. 2024) has been updated.
