# Supplementary material for: Optimization of kidney function in cardiac surgery patients with intra-abdominal hypertension: expert opinion
Source: Perioper Med (Lond). 2024 Jul 12;13:72. doi: 10.1186/s13741-024-00416-5 (PMC11245849; doi:10.1186/s13741-024-00416-5)
Supplement: Supplementary file 1 — Supplementary Material 1: Supplementary Figure S1. Assessment of fluid responsiveness with functional hemodynamics. Supplementary Figure S2. Assessment of abdominal wall compliance (Cab). Supplementary Table S1. When to monitor abdominal wall compliance? Supplementary Table S2. Indicators for decreased abdominal wall compliance. Supplementary Table S3. Indicators for increased abdominal wall compliance. [file 13741_2024_416_MOESM1_ESM.docx]

**Supplemental Information**

Table of Contents

Assessment of fluid responsiveness with functional hemodynamics.…...……..………………... 2

Assessment of abdominal wall compliance.....................................................……....……….…...4

When to monitor abdominal wall compliance *…...……………………………………..*…..……......10

Indicators for decreased abdominal wall compliance……………………………………………11

Indicators for increased abdominal wall compliance…………………………………………….12

References……………………………………….....…………………………………………….13

1. Assessment of fluid responsiveness with functional hemodynamics

1. Passive leg raise test


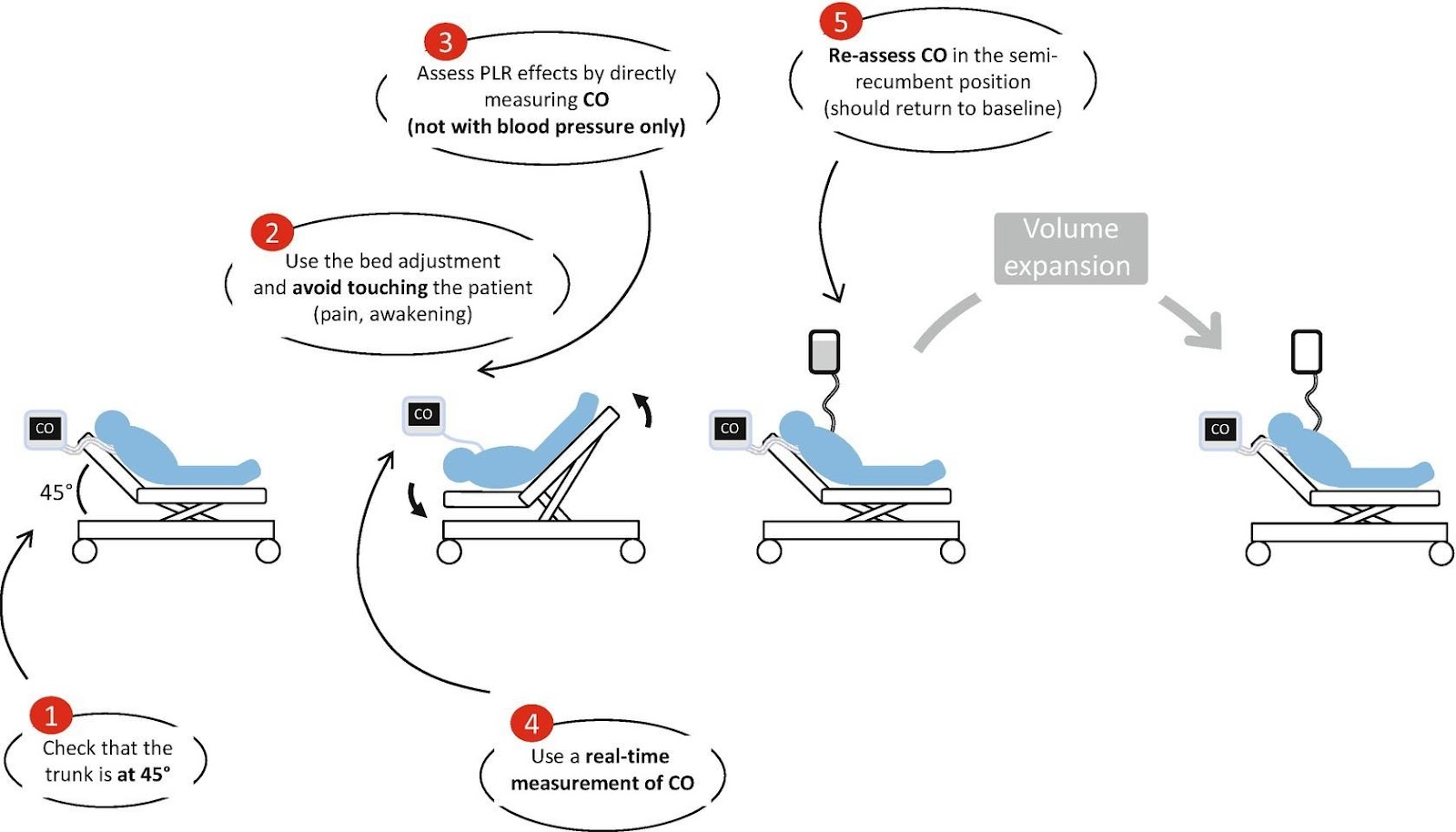


Figure adapted according to open access CC BY Licence 4.0  from Monnet and Teboul with permission (<https://link.springer.com/chapter/10.1007/978-3-030-71752-0_17/figures/2> )

1. Thresholds for fluid responsiveness obtained via different tests


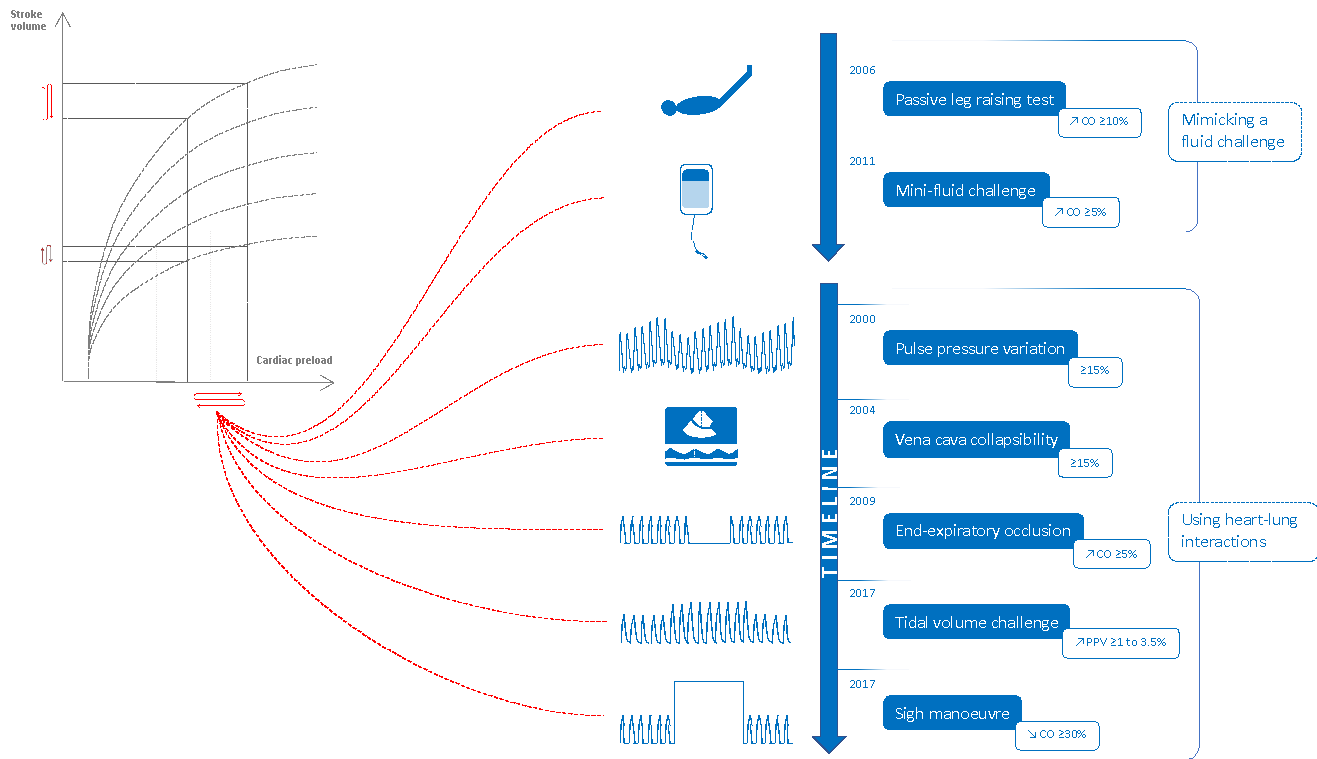


Figure adapted according to open access CC BY Licence 4.0  from Monnet and Teboul with permission (<https://link.springer.com/chapter/10.1007/978-3-030-71752-0_17/figures/2> )

1. **Assessment of abdominal wall compliance (Cab)**
2. *Delta IAP***:** ΔIAP is calculated as the difference between the end-inspiratory (IAP_ei_) and the end-expiratory IAP (IAP_ee_) value:

*ΔIAP = IAP_ei_ – IAP_ee_*


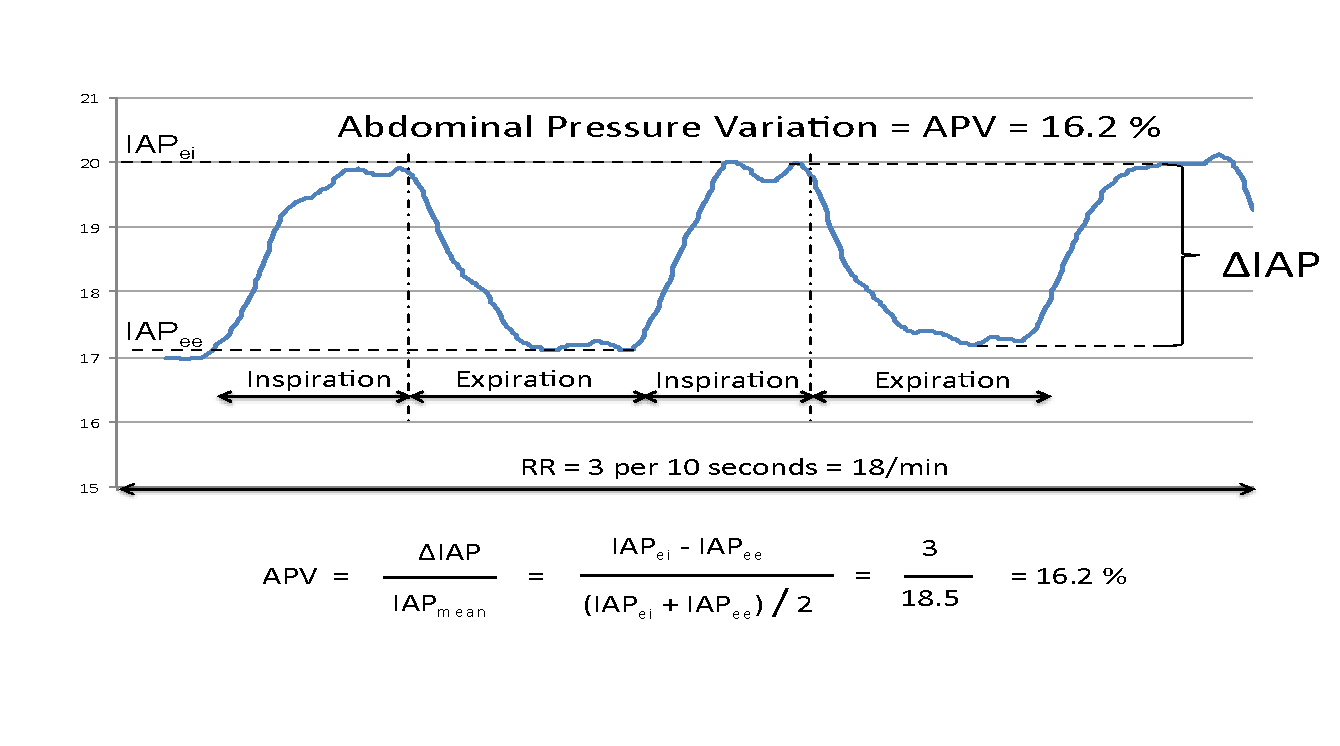


1. *Abdominal pressure variation:*  APV is calculated as the difference between the IAP_ei_ and the IAP_ee_ value or thus ΔIAP divided by the mean IAP (MIAP) and expressed as a percentage:

*APV = ΔIAP/MIAP*

*APV = (IAP_ei_ – IAP_ee_)/MIAP*

1. *IAPmean (MIAP):* The real-time and correct average can be calculated as:

*MIAP = ((Tmax X IAPmax) + (Tmin X IAPmin))/(Ttot = Tmax + Tmin)*

with Tmax the inspiratory time and Tmin the expiratory time – the normal ratio is 1:2 but this can change in ARDS/COVID,… this has been discussed in a paper on mean IAP [1]

or you can also calculate mathematical (maybe would be good to calculate both)

*MIAP = (IAPmax + IAPmin)/2*

**
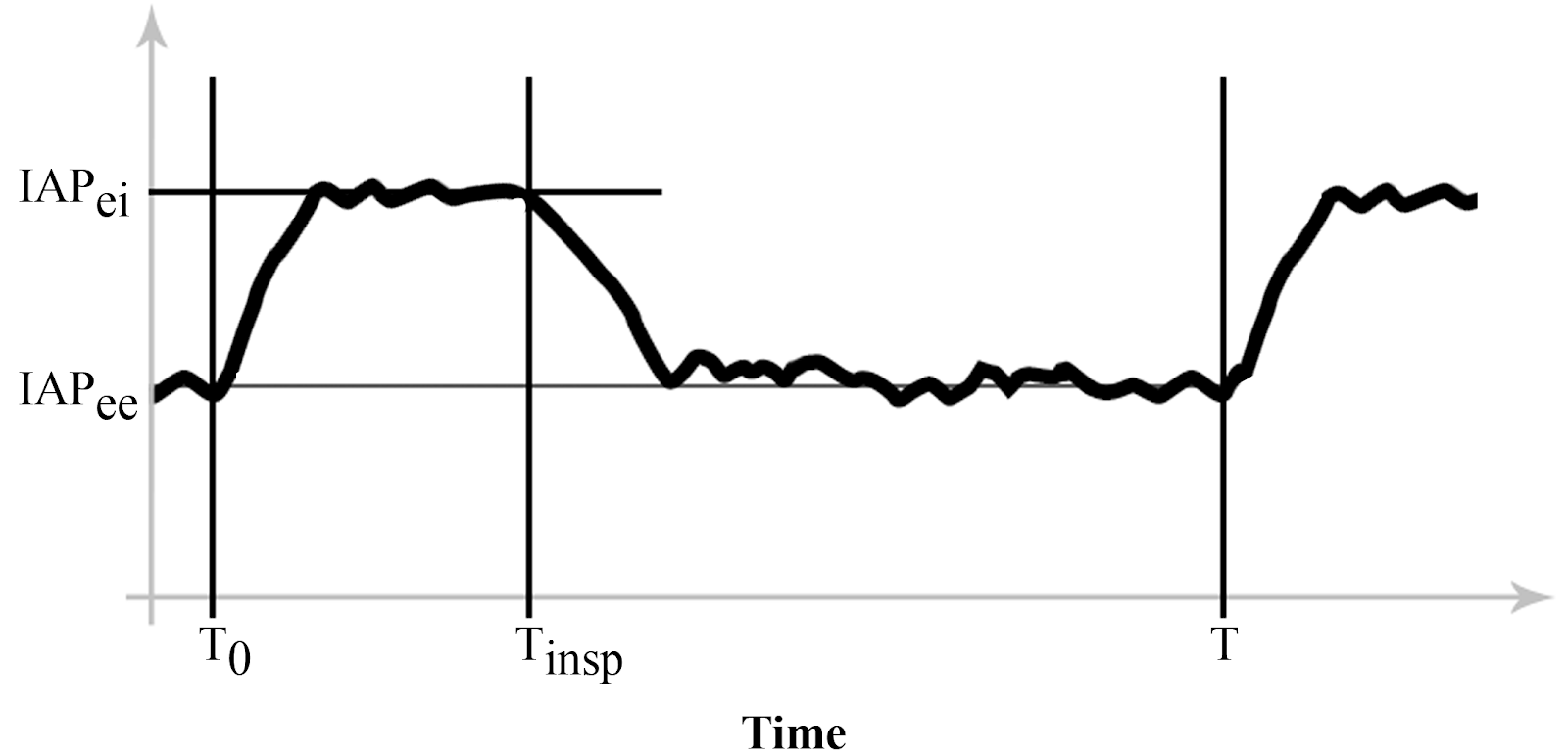
**

1. *Estimation of abdominal compliance during low flow pressure-volume loop*

The Cab can be estimated by analysis of the dynamic changes caused by mechanical ventilation on IAP. During a low flow PV loop to determine the best PEEP one can observe the change in mean IAP (MIAP). The compliance obtained by this manoeuvre can be calculated as follows:

*C_abPV_ = ΔTV/ΔMIAP*

With ΔTV the insufflated volume and ΔIAP the difference between MIAP at the end and start of the PV loop (this is illustrated in Figure)


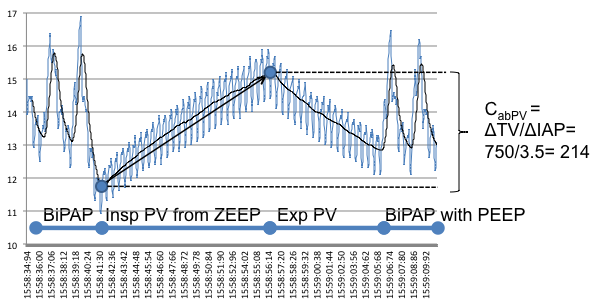


1. *Estimation of abdominal compliance during mechanical ventilation*

Whilst looking at the effects of TV excursions on IAP and by calculating the difference between IAP_ei_ and IAP_ee_ one can also obtain an idea of C_ab_ [2]:

*C_abTV_ =TV/ΔIAP*

The higher the respiratory excursions seen in a continuous IAP tracing, the lower the C_ab_ (for the same TV). The higher the IAP, the higher ΔIAP or thus the lower C_ab_.

1. *Respiratory abdominal variation test (RAVT/IPPV)*

A last non-invasive method for estimation of C_ab_ can be done by performing a respiratory abdominal variation test (RAVT) in IPPV-mode with increasing TV (from 0 to 1000ml with increments of 250ml or 4-6-8-10 ml/kg BW):

*C_abRAVT_ =ΔTV/ΔIAP_ei_*


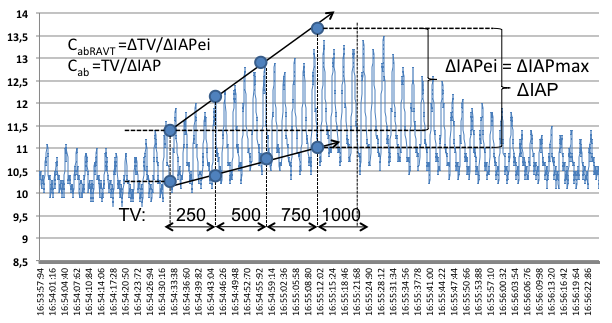


The RAVT can also be performed in BIPAP mode with increasing PEEP levels (from ZEEP to 15 cmH2O) at a certain set IPAP level.

*C_abRAVT_ =ΔTV/ΔIAP_ee_*


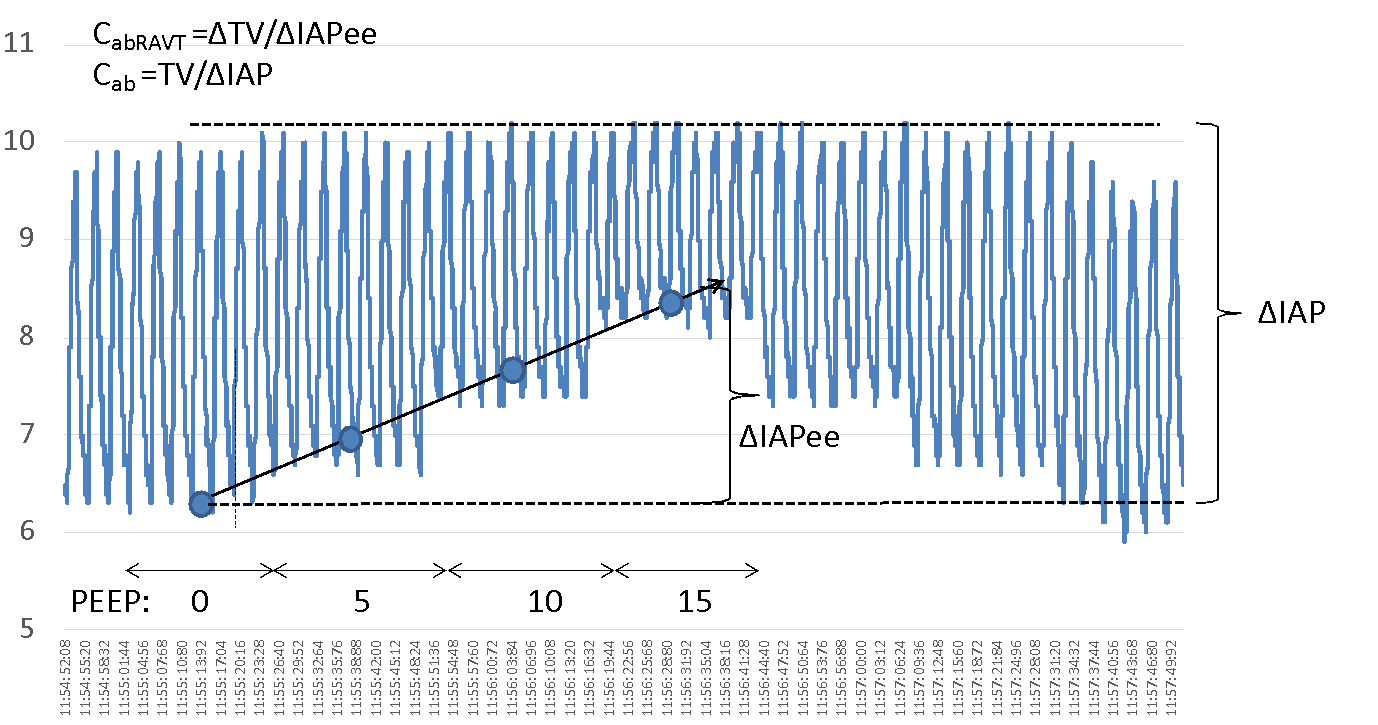


The C_ab_ obtained with RAVT correlates with C_ab_ obtained from ΔIAP during mechanical ventilation [3]. Increasing TV increases IAP_ei_ while increasing PEEP increases IAP_ee_. Future studies should look at the effects of paracentesis or laparoscopy on C_ab_ and ΔIAP to confirm this hypothesis.


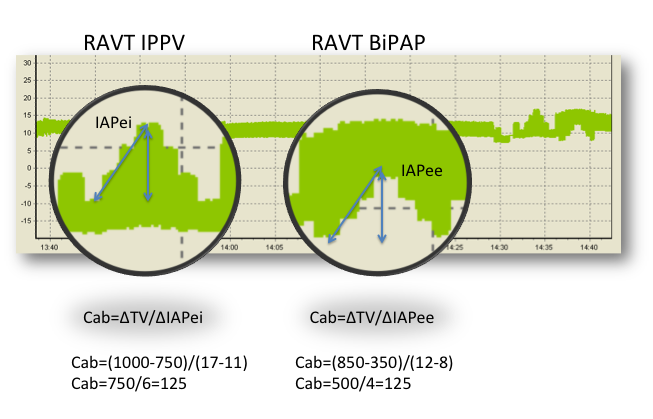


1. *Accommodation of abdominal wall*

Accommodation of the abdominal cavity. Schematic representation of different phases during increasing intraabdominal volume (IAV) in two patients undergoing laparoscopy (CO_2_‐insufflation). Shaded areas represent the reshaping phase (*light blue* – A and A’), the stretching phase (*medium blue* – B and B’) and the pressurization phase (*dark blue* – C and C’). The apostrophe (’) indicates the patient with good abdominal wall compliance. In the patient with poor compliance, the reshaping phase went from an IAV of 0 to 2.8 l (vs 0 to 3.8 l when compliance was normal), the stretching phase from IAV of 2.8 to 5.6 l (vs 3.8 to 7.2 l) and the pressurization phase from > 5.6 l vs 7.2 l in the patient with normal compliance. Adapted from Malbrain et al. with permission [4, 5]


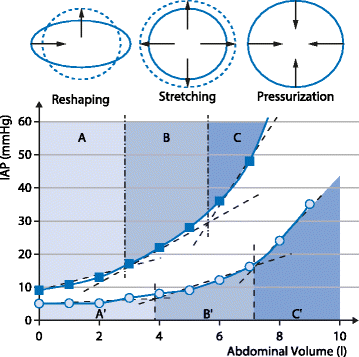


1. *How to calculate transmural CVP?*

CVP_tm_ = CVP_ee_ – ATI x IAP = CVP_ee_ – IAP/2


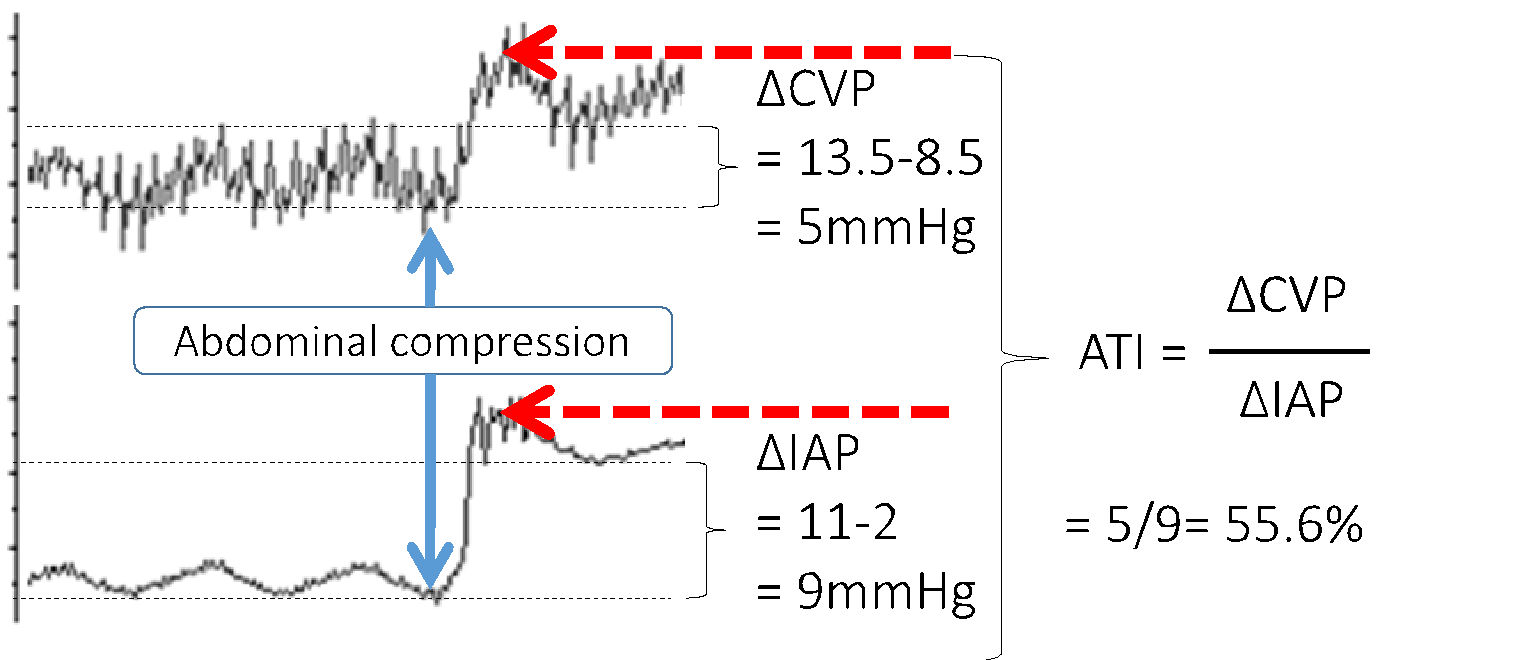


1. **When to monitor abdominal wall compliance?**

| **Related to increased intra-abdominal contents** |
| --- |
| - Gastroparesis |
| - Gastric distention |
| - Ileus |
| - Volvulus |
| - Colonic pseudo-obstruction |
| - Abdominal tumour |
| - Retroperitoneal/ abdominal wall hematoma |
| - Enteral feeding |
| - Intra-abdominal or retroperitoneal tumor |
| - Damage control laparotomy |

| **Related to abdominal fluid, air or blood collections** |
| --- |
| - Liver dysfunction with ascites |
| - Abdominal infection (pancreatitis, peritonitis, abscess,…) |
| - Hemoperitoneum |
| - Pneumoperitoneum |
| - Laparoscopy with excessive inflation pressures - Major trauma |
| - Peritoneal dialysis |

| **Related to capillary leak and fluid resuscitation** |
| --- |
| - Acidosis* (pH below 7.2) |
| - Hypothermia* (< 33°C) |
| - Coagulopathy* |
| - Polytransfusion |
| - Polytrauma - Sepsis |
| - Severe sepsis or bacteremia - Septic shock - Massive fluid resuscitation - Major burns |

1. **Indicators for decreased abdominal wall compliance**

| **Related to antropomorphy and patient demographics** |
| --- |
| - Male gender - Young age (elastic recoil) - Obesity (weight, BMI) - Android composition (sphere, apple shape) - Increased visceral fat - Waist-to-hip ratio >1 - Short stature |

| **Related to comorbidities and/or increased non-compressible IAV** |
| --- |
| - Fluid overload - Bowels filled with fluid - Stomach filled with fluid - Tense ascites - Hepatomegaly - Splenomegaly - Abdominal fluid collections, pseudocyst, abscess - Sepsis, burns, trauma and bleeding (coagulopathy) |

| **Related to abdominal wall and diaphragm** |
| --- |
| - Umbilical hernia repair - Muscle contractions (pain) - Bodybuilders (6-pack abdomen) - IS and anasarca edema (skin) - Burn eschars (circular) - Tight abdominal surgical closure - Abdominal Velcro belt or adhesive drapes - Prone positioning or HOB > 45° - Pneumoperitoneum - Pneumatic anti-shock garments - Abdominal wall bleeding - Rectus sheath hematoma - Correction of large hernias - Gastroschisis, omphalocoele - Mechanical ventilation (IPPV) - Fighting with the ventilator - Use of accessory muscles - Use of positive end expiratory pressure (PEEP) Presence of auto-PEEP (tension pneumothorax) - COPD emphysema (diaphragm flattening) - Basal pleuropneumonia |

1. **Indicators for increased abdominal wall compliance**

| **Related to antropomorphy and patient demographics** |
| --- |
| - Height (tall stature) - Old age (loss of elastic recoil) - Female gender - Lean and slim body - Normal BMI - Gynoid composition (ellipse, pear-shaped) - Waist-to-hip ratio < 0.8 - Peripheral obesity - Preferentially subcutaneous fat |

| **Related to absence of comorbidities and/or increased compressible IAV** |
| --- |
| - No fluid overload - Bowels filled with air - Stomach filled with air - Absence of deadly triad: normothermia, normal pH, normal coagulation |

| **Related to abdominal wall and diaphragm** |
| --- |
| - Umbilical hernia (before repair) - Burn escharotomy (thorax and/or abdomen) - Avoidance of tight closure - Open abdomen with temporary abdominal closure - Beach chair positioning - Sedation and analgesia - Muscle relaxation - Bronchodilation - Lung protective ventilation - Pre-stretching of fascia (cirrhosis with ascites, peritoneal dialysis when fluid is drained from abdomen) - Previous pregnancy - Previous laparoscopy - Previous abdominal surgery - Abdominal wall lift - Weight loss |

1. **References**

1. Ahmadi-Noorbakhsh S, Malbrain ML: Integration of inspiratory and expiratory intra-abdominal pressure: a novel concept looking at mean intra-abdominal pressure. *Ann Intensive Care* 2012, 2 Suppl 1:S18.

2. Sturini E, Saporito A, Sugrue M, Parr MJ, Bishop G, Braschi A: Respiratory variation of intra-abdominal pressure: indirect indicator of abdominal compliance? *Intensive care medicine* 2008, 34(9):1632-1637.

3. Malbrain MLNG, Van deVijver K, Verstraeten AS, Van Regenmortel N, De laet I, Schoonheydt K, Dits H: The Respiratory Abdominal Variation Test (RAVT): A Noninvasive Way To Estimate Abdominal Wall Compliance (cab) At The Bedside. *Am Surg* 2011, 77 (Supplem 1):S98.

4. Malbrain ML, De Laet I, De Waele JJ, Sugrue M, Schachtrupp A, Duchesne J, Van Ramshorst G, De Keulenaer B, Kirkpatrick AW, Ahmadi-Noorbakhsh S *et al*: The role of abdominal compliance, the neglected parameter in critically ill patients - a consensus review of 16. Part 2: measurement techniques and management recommendations. *Anaesthesiol Intensive Ther* 2014, 46(5):406-432.

5. Malbrain ML, Roberts DJ, De Laet I, De Waele JJ, Sugrue M, Schachtrupp A, Duchesne J, Van Ramshorst G, De Keulenaer B, Kirkpatrick AW *et al*: The role of abdominal compliance, the neglected parameter in critically ill patients - a consensus review of 16. Part 1: definitions and pathophysiology. *Anaesthesiol Intensive Ther* 2014, 46(5):392-405.
